# Analysis of Genome-Wide Mutational Dependence in Naturally Evolving *Mycobacterium tuberculosis* Populations

**DOI:** 10.1093/molbev/msad131

**Published:** 2023-06-23

**Authors:** Anna G Green, Roger Vargas, Maximillian G Marin, Luca Freschi, Jiaqi Xie, Maha R Farhat

**Affiliations:** Department of Biomedical Informatics, Harvard Medical School, Boston, MA, USA; Department of Biomedical Informatics, Harvard Medical School, Boston, MA, USA; Center for Computational Biomedicine, Harvard Medical School, Boston, MA, USA; Department of Biomedical Informatics, Harvard Medical School, Boston, MA, USA; Department of Biomedical Informatics, Harvard Medical School, Boston, MA, USA; Department of Genetics, Johns Hopkins School of Medicine, Baltimore, MD, USA; Department of Biomedical Informatics, Harvard Medical School, Boston, MA, USA; Division of Pulmonary and Critical Care Medicine, Massachusetts General Hospital, Boston, MA, USA

**Keywords:** computational biology, microbiology, mycobacterium, microbial genomics, microbial evolution

## Abstract

Pathogenic microorganisms are in a perpetual struggle for survival in changing host environments, where host pressures necessitate changes in pathogen virulence, antibiotic resistance, or transmissibility. The genetic basis of phenotypic adaptation by pathogens is difficult to study in vivo. In this work, we develop a phylogenetic method to detect genetic dependencies that promote pathogen adaptation using 31,428 in vivo sampled *Mycobacterium tuberculosis* genomes, a globally prevalent bacterial pathogen with increasing levels of antibiotic resistance. We find that dependencies between mutations are enriched in antigenic and antibiotic resistance functions and discover 23 mutations that potentiate the development of antibiotic resistance. Between 11% and 92% of resistant strains harbor a dependent mutation acquired after a resistance-conferring variant. We demonstrate the pervasiveness of genetic dependency in adaptation of naturally evolving populations and the utility of the proposed computational approach.

## Introduction

Genomic evolution of pathogenic bacteria is rapid and pervasive and poses a serious threat to global health. The evolutionary pressure imposed by human infection creates pathogens that are more transmissible, more virulent, or more difficult to treat due to antibiotic resistance (Jackson et al. 2011; Farhat et al. 2013; Diehl et al. 2016; Karim and Karim 2021). While often attributed to single mutational events, antibiotic resistance is more complex, and high-level resistance can manifest through multiple mutations in a sequential and dependent manner (Hughes and Andersson 2017; Wong 2017; Kryazhimskiy et al. 2014). Dependency, here defined as when an initial mutation changes the likelihood of a specific subsequent mutation, may arise due to the fitness cost of initial resistance acquisition or the action of antibiotics on multiple cellular processes (Andersson and Hughes 2010; Melnyk et al. 2015). A complete understanding of the multiple, dependent mutations associated with any pathogen phenotype, including resistance, would allow us to better understand pathogen biology and potentially forecast evolution.

Traditionally, the study of mutational dependence in microbial populations has relied on in vitro evolution experiments where populations are longitudinally sampled to determine mutational trajectories (Kryazhimskiy et al. 2014; Safi et al. 2013; Allen et al. 2021; Plucain et al. 2014). This heavily restricts the context and breadth of evolutionary landscapes we can study. Further, resistance acquisition in vitro may not necessarily reflect resistance acquisition in vivo within a host environment. New approaches are needed to understand evolution of natural populations that will necessarily be sampled contemporaneously and be the most relevant to real-world scenarios and human health.

*Mycobacterium tuberculosis* complex (MTBC), the causative agent of tuberculosis, which displays increasing antibiotic resistance globally, is an important case study for identifying mutational dependency (Lange et al. 2018; World Health Organization 2020). Although prior reports have characterized individual cases of dependent evolutionary trajectories in MTBC antibiotic resistance (Safi et al. 2013; Comas et al. 2011; Kavvas et al. 2018), a genome-wide method to detect dependent mutations generalizable to any phenotype is needed. In other bacterial species, recent work has used Potts models and regression with interaction terms to detect dependent evolution in natural populations (Skwark et al. 2017; Puranen et al. 2018; Schubert et al. 2019). However, the strong linkage effects and low diversity of many pathogens, including *M. tuberculosis*, require an alternative approach (Supplementary material online). A well-suited solution to clonally evolving populations is to focus on mutations that evolve in a parallel manner across the phylogeny. This approach has been successful in detecting individual genetic effects on phenotype because it readily controls for population structure, biased sampling, and linkage across the clonal genome (Farhat et al. 2013; Desjardins et al. 2016). Phylogeny-based approaches have successfully found dependencies in influenza proteins (Kryazhimskiy et al. 2011) but have not to date been applied to pairs of mutations in complete bacterial genomes.

**Fig. 1.**
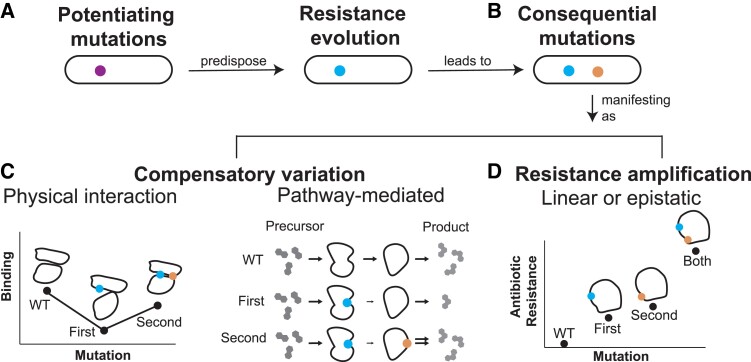
Patterns leading to detected evolutionary dependency. A simple framework classifying observed types of evolutionary dependencies in antibiotic resistance development. (*A*) Dependencies can potentiate resistance development. Potentiating mutations may amplify resistance, that is, directly influence the inhibitory concentration of the drug, or they may instead have a general effect on growth, virulence, and metabolism that increase the probability of acquisition of directly causal drug resistance mutations. (*B*) After initial resistance evolution, consequential mutations (i.e., arising as a consequence of resistance) are observed and manifest through multiple mechanisms. (*C*) Consequential mutations may restore fitness lost with the acquisition of resistance variants. The latter can be mediated through direct physical interactions or pathway-mediated changes in related genes. (*D*) Lastly, consequential mutations can causally amplify resistance, either through individual effects or epistatic effects such that the combination of the two variant effects is different than the sum of the individual effects.

Here, we study pairs of dependent, parallelly occurring (homoplastic) mutations arising during the evolution of natural populations. We determine which mutations are more likely to occur in certain genetic backgrounds, controlling for increased uncertainty when mutations are rare. We applied our method to a data set of 31,428 MTBC genomes spanning six major global lineages, finding that antibiotic resistance and antigen evolution are enriched among dependent mutation pairs. We observe 23 mutations that appear to potentiate the evolution of resistance to multiple different antibiotics. We quantify the number of strains in our data set with evidence of dependent evolution occurring as a consequence of initial resistance evolution to 11 antibiotics—ranging from 92% for streptomycin to 11% for fluoroquinolones. We chart common manifestations of these consequential mutations after antibiotic resistance evolution, finding compensatory variation mediated through both physical interactions and metabolic pathways, and multistep evolution of high-level resistance phenotypes (fig. 1). Overall, our results demonstrate the promise of detecting dependent mutational events in naturally evolving pathogen populations and explore mechanistic explanations for dependencies.

## Results

### Evolutionary Events in *M. tuberculosis*

We estimated the evolutionary history of 31,428 diverse MTBC strains using maximum likelihood phylogeny and ancestral sequence reconstruction, with 2,815, 8,090, 3,398, 16,931, 98, and 96 strains belonging to Lineages 1–6, respectively, as recently described (Vargas et al. 2022; Vargas, Freschi, Spitaleri, et al. 2021) (supplementary Data S1, Supplementary Material online). Restricting our analysis to single-nucleotide polymorphisms (SNPs), we observe 4,743 sites in the genome to have evolved away from the pan-susceptible ancestral state (Comas et al. 2010) at least five times independently (supplementary Data S2, Supplementary Material online). Of these 4,743 sites, 19.5% are intergenic, and the remaining mutations are found in a total of 1,476 different genes. The mutations are well distributed phylogenetically, arising in a median of three major lineages. Most mutations are relatively recent, with a median age index (ratio of number of descendant branches to number of mutation events) of 2.4.

We then categorize the homoplastic mutations in terms of their putative function: labeling mutations as antibiotic resistance associated based on a catalog of known and potential variants (W. H. (hq) Global Tuberculosis Programme 2021) and antigenic based on their presence in proteins with known epitopes (Coscolla et al. 2015; Vita et al. 2019) (Materials and Methods). Antibiotic-associated mutations are overrepresented in our data set of homoplastic mutations, with 5% and 17% of mutations annotated as known or possibly resistance conferring, respectively (chi-squared *P* < 10^−307^ for both) (supplementary Data S3, Supplementary Material online). Homoplastic mutations in epitopes and epitope-containing proteins comprise 3% and 16% of the data set, respectively, again representing a significant enrichment (chi-squared *P* < 10^−67^ and <10^−34^). We find no significant enrichment for homoplastic mutations in essential genes (chi-squared *P* > 0.01). The overrepresentation of antibiotic resistance and antigen-associated homoplastic mutations suggests positive selection for these beneficial traits.

### Detecting Dependencies Between Mutations

Previously, Potts model based methods have been used to detect potential dependencies between mutations in microbial genomes. However, we find that the strong linkage effects in *M. tuberculosis* bias the method toward lineage-defining variants, even after state-of-the-art correction, and therefore we develop an alternative approach (supplementary fig. S1–S3, Supplementary Material online).

We develop a method to detect dependency between pairs of homoplastic mutations. We first partition the data set into two nonmutually exclusive groups: 1) mutation pairs that occur simultaneously on the same branch at least once (*N* = 132,012) and 2) mutation pairs that occur sequentially on subsequent branches at least once (*N* = 998,764).

**Fig. 2.**
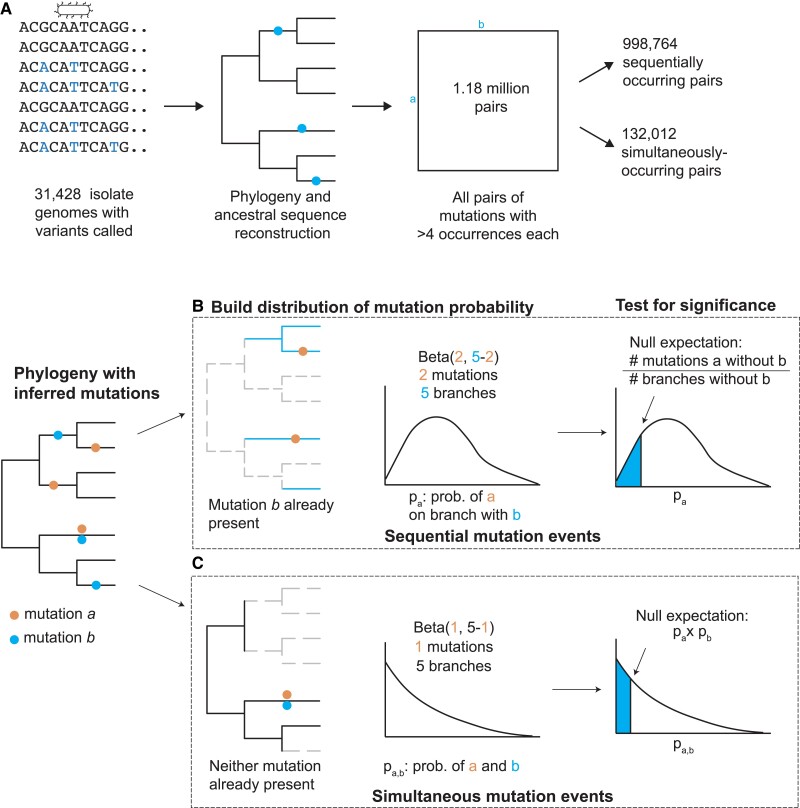
Computational workflow for finding dependencies between mutations. (*A*) We found 1,184,177 pairs of SNPs across 4,743 sites that co-occur either sequentially or simultaneously at least once. We began with a data set of 31,428 isolate genomes and performed phylogeny and ancestral sequence reconstruction. We called each SNP as ancestral or derived relative to the pan-susceptible *M. tuberculosis* ancestral sequence (H37Rv) and then enumerated all SNPs that arise at least five times independently, dividing them into pairs that appear at least once sequentially or simultaneously. (*B*) For sequentially occurring pairs, we determine whether the probability of mutation *a* is affected by the presence of mutation *b* by inferring the distribution of the probability of mutation *a* in the context of *b* using a beta distribution, and then comparing it with the expected probability of mutation *a* not in the context of *b*. (*C*) For simultaneously occurring mutations, we determine whether the probability of observing mutations *a* and *b* simultaneously is higher than expected based on the product of the individual probabilities of mutation *a* and *b*—that is, assuming the two events are independent.

To test for dependencies between sequentially occurring mutations *a* and *b*, we determine if the estimated probability of mutation *a* is higher for a genetic background containing mutation *b* compared with the root ancestral background (fig. 2; Materials and Methods). We exclude pairs of dependent mutations where the median distance between *a* and *b* on the phylogeny is >1,000 mutations, as these represent outliers (Materials and Methods; supplementary fig. S4, Supplementary Material online). We detect significant evolutionary dependency for 3.3% (*N* = 32,567) of all sequentially occurring homoplastic mutation pairs (Benjamini–Hochberg false discovery rate [FDR] < 0.01) (supplementary Data S4, Supplementary Material online).

To test for dependencies between simultaneously occurring mutations *a* and *b*, we determine if the estimated probability of mutations *a* and *b* occurring simultaneously is higher than the estimated frequency of their co-occurrence if the two mutations were independent events (fig. 2; Materials and Methods). We detect significant evolutionary dependency for 48% (*N* = 62,804) of all simultaneously occurring homoplastic mutation pairs (Benjamini–Hochberg FDR < 0.01) (supplementary Data S3, Supplementary Material online). We note the high fraction of significant pairs because simultaneous occurrence of any two mutations on a branch is unlikely.

### Highest-Scoring Sequentially Occurring Dependencies Are Enriched in Antibiotic Resistance Function

We next annotate whether the pairs of sequentially occurring mutations are enriched in antibiotic resistance-associated or antigenic proteins. We greedily assign each pair of mutations into the following categories in the respective order: Both mutations are antibiotic associated, the first or second mutation acquired is antibiotic associated, both mutations are antigenic, one mutation is antigenic, or other (none of the categories apply) (Materials and Methods).

**Fig. 3.**
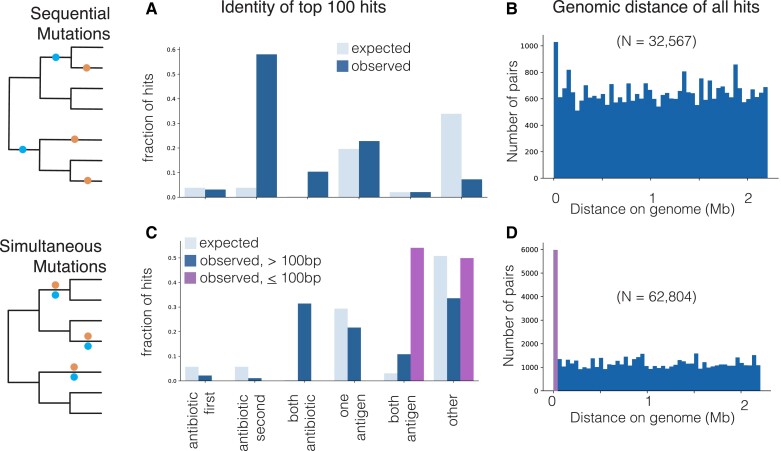
Sequential and simultaneous mutation pairs are enriched in functional categories. We determine the identity of the top 100 pairs of significant hits for (*A*) sequential mutation pairs and (*C*) simultaneous mutation pairs. We categorize mutation pairs as those where a known resistance mutation occurs first, known resistance mutation occurs second, both mutations are known resistance mutations, one mutation is in a known antigen protein, both mutations are in a known antigen protein, or other category (not any of the above). For simultaneous mutations, we compute the categories for the top 100 hits found within 100 bp on the genome and for the top 100 hits found outside 100 bp. The genomic distance in megabases of all pairs of significant dependent mutations for (*B*) sequential mutations and (*D*) simultaneous mutations are shown.

The pairs of sequentially occurring dependent mutations are enriched in antibiotic resistance (actual: 13.6% vs. expected: 10.3%) and antigenic categories (actual: 41.2%. vs. expected: 32.1%) compared with our expectation from the frequencies of individual SNPs (chi-squared value < 10^−307^; supplementary Data S3, Supplementary Material online). This indicates that not only are individual antibiotic resistance and antigen-associated mutations individually under positive selection but that there are relationships between pairs of mutations that render some of them more likely to co-occur in one another's presence. Among the top 100 hits in terms of *P*-value, 59% include a known resistance variant, in the majority of which the resistance-conferring mutation occurs second (fig. 3*A*).

### Enrichment of Simultaneous Mutation Pairs in Close Genomic Proximity

We identify that simultaneously occurring dependent pairs are more likely to be in close genomic proximity than sequentially occurring dependent pairs: Of the top 100 pairs of simultaneous dependencies, 87% are within 100 bp on the genome (fig. 3*B* and *D*).

We investigate the possible origins of the enrichment of these pairs, to determine whether they are the result of selection on epistatic pairs of mutations, or potentially due to non-SNP mutational processes generating more than one mutation at a time. There are 2,361 pairs of dependent simultaneous mutations found within 100 bp on the genome, 15.6% of which are intergenic, somewhat higher than the 9% of the genome that is intergenic (Cole et al. 1998).

We reason that for pairs of mutations with epistasis, we would occasionally observe an individual mutation alone or sequentially rather than simultaneously. Therefore, for the less frequent of the two mutations, we calculate the fraction of the time that it occurs simultaneously with the other mutation versus independently or sequentially. We find that for 395 pairs of mutations, the less frequent mutation occurs <1% of the time independently or sequentially, and for 655 pairs it occurs <20% of the time (supplementary fig. S5, Supplementary Material online). This indicates that there is a subset of significant, simultaneous dependent mutations for which one of the two mutations almost always occurs on the exact same phylogenetic branch as the other mutation. This phenomenon is not explained by mutations in the same codon: <5% of all simultaneous proximal mutations are found in the same codon, and only 17.5% of the 655 proximal, simultaneously occurring, rarely independent or sequential mutations are found in the same codon.

The top five genic hits within 100 bp, in terms of *P* value, are in Rv1945, *esxJ*, Rv1148c, *PPE54*, and *vapC25*. The *esx* and *PE/PPE* gene families have been previously shown to undergo intrachromosomal recombination (i.e., gene conversion) (Uplekar et al. 2011; Karboul et al. 2008; Phelan, Coll, Bergval, et al. 2016), and both Rv1945 and Rv1148c contain a REP13E12 repeat element, which is present in seven copies throughout the *M. tuberculosis* genome and thus presents a possible gene conversion site (Gordon et al. 1999). Finally, *vapC* is a family of toxins with 47 paralogs throughout the genome, presenting another potential site for gene conversion (Ahidjo et al. 2011).

Due to these three lines of evidence—lack of enrichment for genic pairs, a subset of pairs for which the mutations almost never occur independently or sequentially, and high-scoring pairs in genes known to undergo intrachromosomal recombination—we suggest that the enrichment in the number of significant pairs in close genomic proximity is likely driven by non-SNP mutational processes such as intrachromosomal recombination.

### Highest-Scoring Simultaneously Occurring Dependencies Are Enriched in Antigenic and Antibiotic Resistance Function

We find that simultaneously occurring dependent pairs of homoplastic mutations are enriched in functional categories compared with our expectation from the frequencies of individual SNPs (chi-squared *P* < 10^−307^; supplementary Data S3, Supplementary Material online). We examine simultaneously occurring *proximal* mutations (≤100 bp) separately from simultaneously occurring *distant* mutations (>100 bp). Over 50% of the top 100 significant proximal pairs both occur in an antigenic protein (fig. 3*C*). Among the top 100 significant distant pairs, both antigenic and antibiotic resistance-conferring pairs of mutations are overrepresented (chi-squared *P* < 10^−96^).

### Identity of Nonantibiotic Resistance and Nonantigenic Mutation Pairs

We sought to annotate the potential function of the ∼44% (*N* = 41,335) of dependent pairs that do not fall into either resistance-associated or antigenic functional pairs. These 41,335 dependent pairs are constituted of mutations in 3,071 homoplastic sites, 787 of which are intergenic. The top sequential dependency is between Rv2828c, a conserved hypothetical protein, and an intergenic position upstream of transposase Rv2512c. The top simultaneous, nonproximal dependency is between respiratory chain protein NuoJ and probable conserved membrane protein Rv2219A.

We performed a gene ontology (GO) enrichment analysis (Materials and Methods) to determine if certain molecular functions were overrepresented in the set of genes found by our analysis. We find overrepresentation among 46 categories (supplementary Data S7, Supplementary Material online). The top hits include “response to host immune response (GO: 0052572),” which includes a number of PE/PPE proteins, “peptidyl-histidine phosphorylation (GO: 0018106),” constituted of histidine kinase response regulator pairs, and “fatty acid metabolic process (GO: 0006631),” which includes a number of fatty acid—coA ligase (FadD) proteins. As more annotation data for the *M. tuberculosis* genome become available, we hope to be better able to interpret the sequential and simultaneous dependent mutations between other gene categories.

### Potentiating Mutations that Predispose the Evolution of Antibiotic Resistance

We examine whether particular SNPs predispose the evolution of antibiotic resistance, here called potentiator mutations, as these are of high interest for surveillance and genomic prediction. Among all 32,567 pairs of mutations with significant sequentially acquired dependency, the resistance-conferring mutation is second in 3,185. Of these, 1,431 are explained by just 23 initial mutations. We here define these 23 mutations as potentiators because they lead to over 30 different resistance-associated mutations each (table 1), indicating that they do not predispose the strains to the evolution of resistance to a particular antibiotic but rather predispose to resistance phenotypes in general.

**Table 1.** Resistance-Potentiating Mutations Are Associated with Host–Pathogen Interactions.

ßGenomic Position	Gene ID	Gene Name	Possible Gene/Region Function
75233	Intergenic	—	Upstream of possible transcriptional regulator Rv0067c, upstream of possible oxidoreductase Rv0068
340132	Rv0280	*ppe3*	Unknown
454333	Rv0376c	Rv0376c	Unknown
886661	Intergenic	None	Downstream of Rv0792c, upstream of Rv0793
908186	Rv0814c	*sseC2*	Possibly involved in sulfur metabolism (Kapopoulou et al. 2011)
1161026	Rv1038c	*esxJ*	Contains known T-cell epitope (Grotzke et al. 2010)
1287112	Intergenic	—	Upstream of *narG*, downstream of *mutT2*
1340208	Rv1196	*ppe18*	Intracellular survival (Bhat et al. 2012)
1523817	Rv1355c	*moeY*	Molybdopterin biosynthesis protein (Kapopoulou et al. 2011)
1722228	Rv1527c	*pks5*	Mediates surface remodeling (Boritsch et al. 2016)
2122395	Rv1872c	*lldD2*	Promotes survival inside macrophages (Billig et al. 2017)
2338994	Rv2082	Rv2082	Unknown
2626011	Rv2346c	*esxO*	Inferred to increase risk of resistance evolution (Ortiz et al. 2021), promotes survival inside macrophages (Mohanty et al. 2016)
2626108	Rv2346c	*esxO*	Promotes survival inside macrophages (Mohanty et al. 2016)
2626189	Intergenic	—	Upstream of *esxO*
2626191	Intergenic	—	Upstream of *esxO*
2867575	Rv2544	*lppB*	Unknown
3446699	Rv3081	Rv3081	Unknown
3482717	Intergenic	None	Downstream of Rv3115, Upstream of molybdopterin Cofactor biosynthesis protein MoeB2
3894732	Rv3478	*ppe60*	Host immune response (Su et al. 2018)
4046007	Rv3603c	Rv3603c	Conserved hypothetical alanine- and leucine-rich protein
4060588	Rv362 °c	*esxW*	Influencing increased transmission in Beijing lineage (Holt et al. 2018)
4338371	Rv3862c	*whiB6*	Transcriptional regulator with known role in kanamycin resistance (Zhang et al. 2013; Farhat, Freschi et al. 2019), may modulate virulence (Chen et al. 2016)

Note.—Genomic position, identifier, and name for each of 23 mutations found to occur before at least 30 different resistance-conferring mutations. We include a known or possible function for each gene and intergenic region, if one exists, focusing on possible roles in resistance evolution and host adaptation.

We discover several previously implicated SNPs among our antibiotic potentiators. This includes position G2626011A (EsxO I54I*)*, previously found to increase the risk of resistance evolution (Ortiz et al. 2021), and three other SNPs in the *esxO* gene body or upstream region. We also identify mutations in proteins known to increase intracellular survival of *M. tuberculosis*, G1340208A (PPE18 R287Q) and C2122395T (LldD2 V253M*)*, and a mutation previously associated with increased transmission in the Beijing lineage, T4060588C (EsxW T2A), to potentiate resistance (Bhat et al. 2012; Billig et al. 2017; Holt et al. 2018).

### Consequential Mutations That Compensate for or Amplify Antibiotic Resistance

**Fig. 4.**
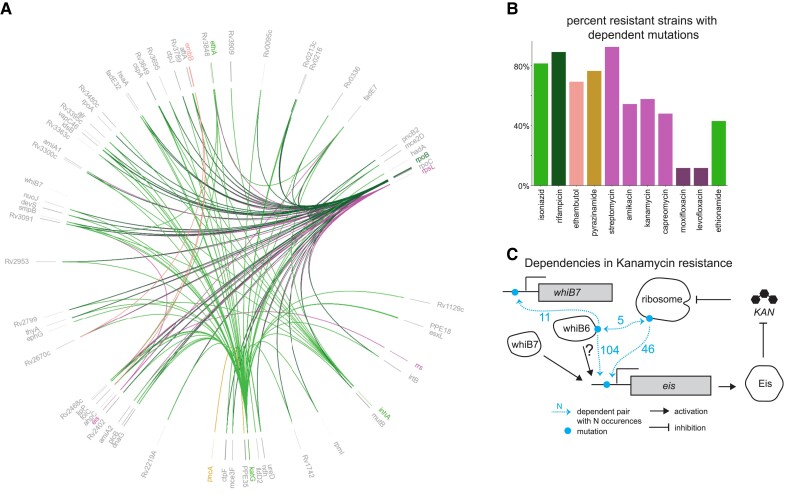
Dependent mutations within resistance-associated genes. We measured the identity and prevalence of significant dependent mutations occurring after initial resistance evolution. (*A*) Mutations that occur after mutations in known antibiotic resistance genes, visualized on the genome using pyCircos (github.com/ponnhide/pyCircos), with colors corresponding to the antibiotics in *B*. Antibiotics with shared genetic basis of resistance are shown in the same color. Only mutations that happen sequentially at least five times are shown. (*B*) Fraction of resistant strains that display one or more pairs of sequential dependent mutations. (*C*) Example of pairs of dependent mutations within the kanamycin resistance pathway, shown on a per-gene basis. Kanamycin's inhibition of the ribosome is blunted by ribosomal RNA mutations, while cellular kanamycin levels are reduced by increased levels of Eis, putatively caused by both mutations in the *eis* promoter region and mutations in the regulatory proteins WhiB7 and WhiB6. Dependencies between these mutations demonstrate multistep resistance evolution.

We next focus on dependent mutations occurring as a consequence of the initial evolution of resistance, here called consequential mutations, since these may indicate potential new mechanisms of resistance evolution or compensation for loss of fitness from initial resistance mutations. We detect 3,724 significant dependent mutations after initial resistance mutations, 1,879 of which are not previously indicated to be involved in resistance, with hits for all 11 antibiotics (supplementary Data S6, Supplementary Material online). We quantified the prevalence of evolutionarily dependent mutations in resistant isolates in our data set (Materials and Methods). We found that a substantial percentage of strains with initial resistance-causing mutations have sequentially acquired dependencies, ranging from 92% for streptomycin to 11% for fluoroquinolones, indicating a pervasive role in antibiotic resistance evolution (fig. 4).

As a positive control, the most frequent consequential mutation we detect is the known dependency between rifampicin resistance mutations in RNA polymerase *β* subunit (RpoB) and substitutions in the RNA polymerase *β*′ subunit (RpoC), which compensate for the loss of fitness incurred by RpoB mutations through a direct physical interaction (Comas et al. 2011). We also detect dependency between the catalase–peroxidase KatG and position G2726142A, in the *ahpC* gene promoter. Increased levels of the AhpC protein are recognized to compensate for the loss of KatG peroxidase activity (Gygli et al. 2017; Ramaswamy et al. 2003), demonstrating a possible case of compensatory substitutions mediated by metabolic pathways. The detection of these known relationships reinforces the utility of phylogenetic methods in reconstructing evolutionary dependency.

Our method also detects new relationships. For the aminoglycoside antibiotic kanamycin, we observe consequential mutations likely resulting in amplification of antibiotic resistance between the 16S rRNA gene *rrs*, the target of kanamycin; sites in the promoter region of the N-acetyltransferase gene *eis*, known to degrade kanamycin (Zaunbrecher et al. 2009; Chen et al. 2011); sites upstream of the transcriptional regulator *whiB7*, known to influence *eis* transcription (Reeves et al. 2013); and sites in the transcriptional regulator *whiB6* (fig. 4). Our findings and previous association studies suggest a role for WhiB6 in kanamycin resistance (Zhang et al. 2013; Farhat, Freschi, et al. 2019). The observed evolutionary dependency suggests that multiple mutations are required to amplify resistance to a high level—mutations in *rrs* disrupt kanamycin binding, while mutations in *whiB6*, *whiB7*, and *eis* likely increase levels of the Eis protein, leading to increased kanamycin degradation.

A GO analysis identified significant enrichment of mutations in proteins from 123 GO categories following the evolution of antibiotic resistance (Materials and Methods) (supplementary Data S7, Supplementary Material online) (Gene Ontology Consortium 2021; Mi et al. 2019; Ashburner et al. 2000). One of the top categories is “regulation of DNA-templated transcription elongation,” of major interest since the first-line antibiotic rifampicin targets the RNA polymerase. We find that the RNA polymerase termination factor *nusG* is repeatedly mutated after initial evolution of rifampicin resistance. NusG is notable because it binds directly to the RNA polymerase subunit RpoB (Said et al. 2021), the target of the drug rifampicin (Farhat, Sixsmith, et al. 2019). The mutated position in NusG, R124H/L, is found at the NusG–RpoB interface (Materials and Methods), suggesting that it is involved in stabilizing the action of the mutated polymerase, similar to the compensatory relationship between RpoC and RpoB (Comas et al. 2011) (fig. 4).

A frequent mutation to follow antibiotic resistance in our data set is HadA C61S, which occurs 40 independent times sequentially or simultaneously with isoniazid resistance evolution and is found in all four major lineages. This mutation is known to confer resistance to the now-obsolete antibiotics thioacetazone and isoxyl (Gannoun-Zaki et al. 2013; Dover et al. 2007) and to candidate new antibiotics (Dover et al. 2007; Dong et al. 2015). Although the observed HadA mutations are potentially attributable to historical coadministration of thioacetazone and isoniazid (Okwera et al. 1994), and hence sequential selective pressure, they may also be consequential mutations of isoniazid resistance—HadA is upstream of the isoniazid drug target InhA in the mycolic acid biosynthesis pathway (Vilchèze 2020) and may play a role in amplifying isoniazid resistance levels or compensating for InhA mutations.

### Sequential Environmental Pressures Lead to Evolutionary Dependency in Antibiotic Resistance

**Fig. 5.**
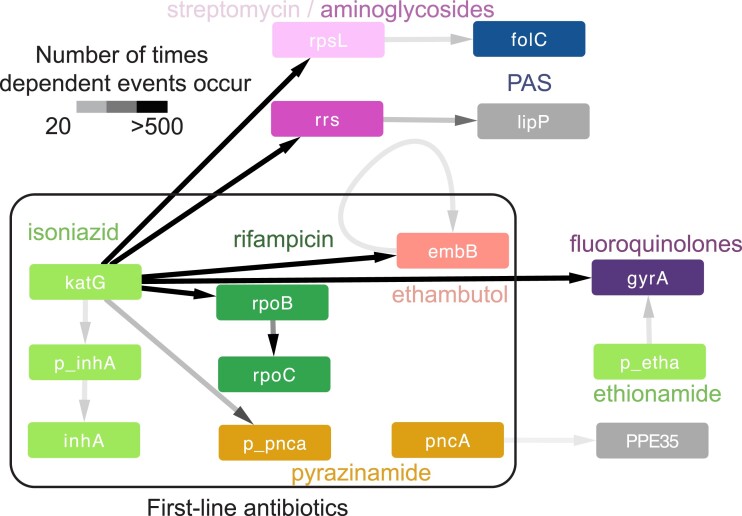
Dependencies between antibiotics. The detected significant dependent mutations between resistance-conferring mutations follow a particular order that mirrors the usage of different antibiotics. For each antibiotic, we took the top dependent pair between known resistance-conferring genes and other genes and between known resistance genes for different antibiotics. We display pairs and links where mutation *a* occurs sequentially or simultaneously with mutation *b* at least ten times. Link intensity corresponds to the number of occurrences. The prefix “p_” before a gene name indicates that the mutations are found in the upstream region of that gene. The drug para-aminosalicylic acid (PAS) is not included in the WHO catalog, but *folC* is a candidate resistance gene for this drug (Wei et al. 2019).

In natural populations, several environmental pressures may act contemporaneously on a population. For pathogenic bacteria, this can take the form of simultaneous or sequential administration of antibiotics to achieve cure. We find strong dependencies between mutations that confer resistance to different antibiotics (fig. 5*A*). Notably, this recapitulates the ordering of antibiotic administration in therapy: Second-line drug resistance-conferring mutations were consistently acquired on a background of resistance to first-line agents (40,054 times a significant sequential mutation event proceeds from first line to second line, vs. 4,310 times they proceed from second line to first line) (supplementary fig. S6, Supplementary Material online). The observed dependencies also confirm postulated relative fitness costs of resistance mutations for the four first-line drugs (Manson et al. 2017; Ektefaie et al. 2021). These findings demonstrate that evolutionary dependency can be used to study not only molecular dependencies that amplify or stabilize a particular phenotype but also environmental forces when the genetic underpinnings of adaptation are known.

### Measuring the Effect of Dependent Mutations on Resistance Phenotypes

We tested whether the observed dependent mutations can be measured, either linearly or epistatically, to have a detectable influence on antibiotic minimum inhibitory concentrations (MICs) by implementing linear mixed models in GEMMA (Materials and Methods) (Zhou and Stephens 2012). The percent of dependent events with a detectable influence on MIC, either linearly or epistatically, ranged from 3% for pyrazinamide to 29% for moxifloxacin, with a median of 9% (supplementary table S1, Supplementary Material online). Notable examples include a promoter variant in position 4243217 in the *embCAB* locus with a positive linear influence on ethambutol MIC, and a synonymous variant in position 332951 (VapC25 P62P) with a measure positive epistatic influence on rifampicin resistance (supplementary table S2 and supplementary Data S8, Supplementary material online). VapC25 is a toxin suggested to promote antibiotic tolerance by slowing growth rate in host (Winther et al. 2016). We observe that 74% of the 23 potentiator mutations have a positive, epistatic influence on MIC for at least one drug. We suggest that at present, the power of linear mixed modeling approaches to detect influences on MIC is limited—currently only a median of 27% of known resistance-conferring mutations were determined to have a detectable statistical influence on resistance, indicating that greater power is needed to detect all effects (supplementary table S1, Supplementary Material online), and therefore expect more dependent mutations to have a detectable effect as more data become available.

We then examined heritability of MIC and the proportion of variance explained (PVE) by the dependent mutations using a series of antibiotic-specific linear mixed models (with up to *n* = 1,469) observations. MIC is a trait with high heritability—previously estimated at 64–88% per drug based on all sites in the genome (Farhat, Freschi, et al. 2019). Compared with heritability estimated from all homoplastic sites, heritability explained by mutations in known or suspected resistance conferring-genes has a median deficit of 32% per antibiotic (table 2). Incorporating sites found to have mutation dependencies with antibiotic resistance genes resulted in a median increase of 24% in heritability, accounting for much of the deficit in heritability using only 37% as many sites, despite these dependencies having been derived without phenotypic data. This demonstrates that our proposed mutational dependency analysis is evolutionarily meaningful and characterizes the genetic architecture of antibiotic resistance phenotypes, even if the current analyses lack the power to detect individual sites and pairs as significantly influencing phenotype in a regression analysis.

**Table 2.** Incorporating Dependent Mutations Explains Heritability of Antibiotic Resistance.

Drug	Homoplastic Sites			Resistance Genes				Resistance Genes and Dependent Pairs			
	PVE	SE	*N* sites	PVE	SE	*N* sites	PVE Difference	PVE	SE	*N* sites	PVE Difference
Amikacin	0.70	0.03	2,013	0.50	0.05	102	0.20	0.53	0.04	739	0.03
Capreomycin	0.54	0.05	1,665	0.28	0.05	93	0.26	0.39	0.05	578	0.11
Ethambutol	0.59	0.04	2,389	0.27	0.04	76	0.32	0.46	0.04	854	0.19
Ethionamide	0.55	0.05	1,920	0.12	0.03	61	0.43	0.48	0.06	389	0.36
Isoniazid	0.71	0.03	2,393	0.22	0.04	69	0.49	0.68	0.03	602	0.46
Kanamycin	0.70	0.03	1,921	0.51	0.06	68	0.19	0.61	0.04	705	0.1
Moxifloxacin	0.69	0.03	1,761	0.32	0.07	28	0.37	0.6	0.05	419	0.28
Pyrazinamide	0.62	0.04	1,727	0.65	0.05	157	−0.02	0.61	0.06	1,338	−0.04
Rifampicin	0.66	0.03	2,437	0.31	0.04	152	0.35	0.58	0.04	964	0.27
Streptomycin	0.59	0.03	2,397	0.33	0.04	211	0.26	0.41	0.04	1,180	0.08
Median	0.64	0.03	1,967	0.32	0.05	84.50	0.32	0.56	0.04	722	0.24

Note.—We compute the heritability (PVE) and standard error (SE) of antibiotic MIC using 1) all homoplastic sites in our data set, 2) homoplastic mutations in known and suspected resistance conferring sites, and 3) homoplastic mutations in known and suspected resistance conferring sites, as well as mutations found to be dependent with known resistance conferring mutations (single sites and interaction terms). Note that “*N* sites” refers to the number of sites included in the analysis that were actually found to have a polymorphism in the isolates with MIC available.

## Discussion

We propose a new method to uncover evolutionary dependencies between mutations in naturally evolving populations and apply it to 31,428 isolates of MTBC. We find both sequentially and simultaneously occurring pairs of dependent mutations, which are enriched in antibiotic resistance and antigenic function. We detect 23 potentiating mutations that predispose the evolution of resistance mutations to several antibiotics and also have a measurable statistical interaction on antibiotic MICs in regression models. We also explore consequential mutations that are acquired in a dependent manner subsequent to resistance acquisition, providing possible examples of novel pathway-mediated selection. We lastly demonstrate the power of this approach in capturing environmental dependencies when the genetic mechanisms are well understood.

We observe that simultaneously occurring mutations are enriched in pairs of mutations within 100 bp on the genome. These mutations rarely occur independently or sequentially, are not enriched in coding sequences, and the top scoring pairs are in genes previously shown to undergo recombination (Uplekar et al. 2011; Karboul et al. 2008). We suggest that the enrichment of simultaneous pairs in close genomic proximity is due to non-SNP mutational processes such as intrachromosomal recombination (gene conversion), which could simultaneously introduce multiple variants in close proximity. Gene conversion has been previously postulated to drive *esx* gene evolution, which are genes enriched in antigenic function (Uplekar et al. 2011). Furthermore, our observed length scale of potential recombination events (<600 bp) matches the observations of RecA-mediated recombination tract length in other species (Santoyo et al. 2005). Innovatively, our results suggest that other genes and especially antigenic genes may evolve through gene conversion, but this requires further validation, potentially with long-read sequencing data. After excluding the proximal dependencies, simultaneous distant dependencies are also enriched in antigenic function and in antibiotic resistance. The observation of simultaneous acquisition of antibiotic resistance pairs of variants may relate to the phylogenies' inability to temporally resolve the two events due to sparse sampling or due to rapid acquisition of the phenotypes in time.

We examine dependent mutations that arise before antibiotic resistance, here called potentiating mutations, or after antibiotic resistance, here called consequential mutations. Consequential mutations appear to fall into at least two categories, those that compensate for loss of fitness due to resistance acquisition and those that amplify the phenotype of antibiotic resistance itself. For example, *nusG* mutations appear to compensate for destabilizing *rpoB* mutants based on our structure analysis, and *hadA* knockdowns were found to significantly sensitize strains to high levels of isoniazid in a recent CRISPRi study (though the magnitude of depletion was below study threshold) (Li et al. 2022). In contrast, the mechanism by which our 23 observed potentiating mutations predispose the evolution of antibiotic resistance is still in question. One possibility is that proteins on the cell surface, including antigenic proteins, play a direct role in antibiotic resistance, for example by altering cell permeability. This possibility is supported by the observation that 74% of observed potentiating mutations have a detectable epistatic influence on MIC. Another possibility is that strains with potentiating mutations may be more likely to transmit between hosts or progress from latent to active tuberculosis disease, leading to higher exposure to antibiotic treatment. Strains with potentiating mutations may also reach higher effective population sizes within host, leading to higher probability of resistance evolution. Finally, strains with potentiating mutations may have higher overall fitness, preemptively compensating for loss of fitness due to resistance evolution.

We investigated whether the detected dependencies were associated with higher antibiotic resistance levels as measured by strain MICs. Dependent mutations when added to known resistance-conferring variants capture the majority of heritability, and several mutations including 74% of the 23 potentiating mutations have measurable associations on resistance. As more MIC data become available, we expect that the power of these analyses to capture the individual effects of dependent mutations will improve.

Our method relies on repeated observations of evolutionary events to infer significant nonindependence of mutations. Therefore, its power is dependent on the number of times a mutation has arisen and thus is biased against the effects of very recent selection, for example responses to newer antibiotics, such as linezolid, clofazimine, and even fluoroquinolones. The smaller numbers of dependent mutations observed for these drugs should not be taken as an assertion that there are fewer dependent mutations as a result of the evolution of resistance to these drugs but rather that we do not yet have enough observations of evolutionary trajectories to reliably infer significance. This issue is also present in the case of pyrazinamide, where a large number of variants in the *pncA* gene are known to cause resistance, and thus the statistical signal is diluted over a large number of variants. A future extension to address this limitation is the expansion to study dependence between mutational burden measured per gene or regions.

The links inferred by our method are based only on the presence of pairs of mutations and thus capture associations both due to true dependency and due to other forces that generate similar patterns. One such force is simultaneous and/or sequential environmental pressures, such as the sequential use of antibiotic treatments. Another possible cause of dependency is transitivity, where mutation *c* is dependent on *b*, which is dependent on *a*, leading to apparently dependency of *c* on *a*. A final possibility is differences in the rate of occurrence of two mutations—if *a* and *b* have the same fitness effect, but *b* is more likely to occur, it will tend to reach fixation first. Therefore, the pairs discovered in this manuscript require further investigation, ideally through experimental or computational association with phenotype, to determine the cause of their manifested dependency.

We believe the method introduced here will be readily generalizable to other microbial species. While *M. tuberculosis* generally does not participate in horizontal gene transfer and thus our method focused on SNPs, our framework could extend to analyzing not just the probability of individual mutations but the probability of gene acquisition or other mutation events. Our method has broad conceptual applicability to understanding clonal evolution ranging from viruses to cancer cells. We show that in *M. tuberculosis*, dependent mutational events are enriched in mutations associated with antibiotic resistance and antigenic function. We discover 23 mutational events that appear to potentiate antibiotic resistance, and dependent events arising as a consequence of resistance are due to both compensatory variation and amplification of resistance phenotypes. Together, these results represent a wealth of new knowledge about the evolution of an important microbial pathogen.

## Materials and Methods

### Data Set of Variable Positions in *M. tuberculosis* Strains

We use a previously curated data set of 782,565 positions with SNPs in any of 31,428 *M. tuberculosis* isolates, from Vargas, Freschi, Spitaleri, et al. (2021) (*Antimicrob Agents Chemother*) (supplementary Data S1, Supplementary Material online). These isolates represent six major *M. tuberculosis* lineages, in which whole-genome sequence data were processed using a previously validated pipeline (Ezewudo et al. 2018; Freschi et al. 2021). Briefly, reads are aligned to the H37Rv reference genome (Cole et al. 1998) using BWA-MEM v0.7.17 after trimming and filtering with PRINSEQ v0.20.4 and contaminant removal with Kraken v0.10.6 (Schmieder and Edwards 2011; Wood and Salzberg 2014; Li, 2013). Variant calling is performed with Pilon v1.2.2, and duplicate reads were removed using Picard v2.9.2 (Walker et al. 2014; Picard toolkit 2019). All isolates had at least 95% of bases with a minimum of ten times coverage after mapping to the reference genome.

To remove low-quality SNPs, we required every SNP to meet all of the following criteria, as originally outlined in Vargas, Freschi, Spitaleri, et al. (2021): 1) The call was designated as *Pass* by Pilon; 2) the mean base quality was >20; 3) the mean mapping quality was >30; 4) none of the aligned reads supported an indel; 5) there was a minimum coverage of 20 reads at the position; and 6) at least 75% of the reads aligning to that position supported a single allele—that is, the position did not have a mixed allele call. The list of 782,565 positions comprises all positions with a SNP relative to the H37Rv reference, after removing positions found in mobile genetic element regions (e.g., transposases, integrases, phages, or insertion sequences) (Comas et al. 2010; Vargas, Freschi, Marin, et al. 2021), found in overlapping genes, or with missing calls in >10% of isolates.

### Reconstructing Mutational History

We used a prior data set that reconstructed the evolutionary history of the 782,565 positions from 31,428 genomes by constructing phylogenetic trees and performing ancestral sequence reconstruction (Vargas et al. 2022). Briefly, phylogenetic trees were constructed based on the variable positions for each lineage using IQ-TREE, using *Mycobacterium canettii* as an outgroup (Nguyen et al. 2015). Trees were constructed independently for each lineage due to memory constraints. For Lineages 1–4, the substitution model used was GTR + F + I + R. For Lineages 5 and 6, which had many fewer representatives, automatic model selection with ModelFinder Plus was implemented (Kalyaanamoorthy et al. 2017). Ancestral sequence reconstruction was performed using SNPPar v.1.0 with options: –-sorting intermediate –-no_all_calls —-no_homoplasic (Edwards et al. 2020).

### Selecting Mutations for Dependency Analysis

We annotate each SNP as either *to* or *from* the ancestral state based on an inferred ancestor of extant the MTBC (Comas et al. 2010). For position 2030521, in the *esxM* gene, the ancestral sequence is inferred to be “A,” but because none of the extract strains have an “A” in this position, we replace the ancestral nucleotide with the most common genotype at this position, “T.” In order to reduce computational time and focus on sites under the strongest positive selection, we selected only those sites that are mutated away from the ancestral state at least five times. To ensure that inferred mutational events are not sequencing errors, we remove sites based on empirical base-pair recall (EBR < 0.90) using the table 210112_EBR_V7_36CI.npz from https://github.com/farhat-lab/mtb-illumina-wgs-evaluation/, download date December 11, 2022. We also remove sites designated as Illumina blindspots by Modlin et al. (2021) (supplementary table S7, Supplementary Material online). This resulted in a total of 4,743 sites.

### Designating Sequentially and Simultaneously Occurring Mutation Pairs

To study mutational dependencies, we consider nonancestral pairs of mutations that occurred either sequentially or simultaneously. Simultaneous mutations are pairs inferred to have occurred on the same branch of the tree (note that these mutations may not have actually occurred simultaneously in a single mutation event, but their ordering cannot be resolved). Sequential mutations are pairs are inferred to have occurred on different, sequential branches.

To accomplish this, we construct a matrix of mutation events with dimensions N (number of branches in all trees, 62,846) by P (number of sites considered, 4,743). This matrix contains a 1 if a particular mutation ***p*** is inferred to occur on a particular branch ***n***, and a 0 otherwise. We also construct a background matrix with dimensions N by P, which contains a 1 if a particular mutation ***p*** is inferred to occur or already have occurred (i.e., be present in the strain genetic background) on branch ***n***, and a 0 otherwise. Python 3.9.13, ETE3 v3.1.2, and NumPy v1.23.1 were used to process tree data into matrices (Huerta-Cepas et al. 2016; Harris et al. 2020).

We enumerated all pairs of simultaneous mutations by comparing columns of the mutation event matrix, to find pairs of sites with mutations that occur on the same branch, for a total of 132,012. We enumerated all pairs of sequential mutations by comparing the mutation event matrix with the background matrix, to find all pairs of sites where one mutation event occurs on the genetic background of another event, for a total of 1,184,177 pairs. Note that a pair may be both simultaneous and sequentially occurring. For sequential pairs, a dependency between position ***a*** and position ***b*** is not equivalent to one between ***b*** and ***a***, and therefore we keep track of these pairs separately. For simultaneous pairs, a dependency between position ***a*** and position ***b*** is equivalent to one between ***b*** and ***a***, and therefore we only keep track of one pair.

### A Model to Detect Evolutionary Dependency Between Sequentially Occurring Mutation Pairs

We next seek to test whether the sequentially occurring mutational events are occurring more frequently than expected—that is, displaying some form of dependency. We model the probability of a given nonancestral mutation, ***a***, in the presence or absence of a second mutation, ***b***, as follows: In the phylogenetic tree with N branches, we define the Bernoulli random variable *X* to indicate whether a mutation occurs on a particular branch. For example, *X_a,n_* = 1 if ***a*** evolves on the ***n***th branch and *X_a,n_* = 0 if mutation ***a*** does not occur on the ***n***th branch. We define the Bernoulli random variable *Y* to indicate whether a mutation has already evolved prior to a particular branch. For example, *Y_b,n_* = 1 if ***b*** evolved prior to the ***n***th branch, and *Y_b,n_* = 0 if ***b*** did not evolve prior to the ***n***th branch.

We model the probability of ***a***, *P*(*X_a,n_*), as a beta distribution, the conjugate prior of the Bernoulli distribution. The shape parameters *α* and *β* of the beta distribution are given by the count of observed branches where *X_a,n_* = 1 and *X_a,n_* = 0, respectively:


$$\alpha =\sum_{n=1}^{N}X_{a,n}\;$$

$$\beta =N-\alpha -\sum_{n=1}^{N}Y_{a,n}$$

Branches where mutation *a* has already occurred (i.e.*, Y_a,n_* = 1) are subtracted because there is no possibility of further mutation in our model. Because most branches in our phylogeny are short (72% have ten or fewer mutations, and 99% have 100 or fewer mutations, supplementary fig. S4, Supplementary Material online), we do not consider branch lengths in our analysis.

Because we seek to test whether *P*(*X_a_*|*Y_b_* = 1) is different from *P*(*X_a_*|*Y_b_* = 0), we partition the branches into two sets: those with mutation ***b***, {*L* |*Y_b_*_,l_ = 1 or *X_b,n_* = 1}, and those without mutation ***b*** {*M* |*Y_b,m_* = 0 and *X_b,n_* = 0}. To test whether the two distributions are different, we test the hypothesis that the expected value of Beta(*α_M_*, *β_M_*) is drawn from Beta(*α_L_*, *β_L_*) by computing the *P* value. This approach to modeling *P*(*X_a_*) using the observed mutation data captures the higher uncertainty about *P*(*X_a_*|*Y_b_* = 1) when the number of branches in {*L*} is small, because the variance of the beta distribution is higher for smaller values of *α* and *β*.

### Detecting Evolutionary Dependency Between Simultaneously Occurring Mutations

To determine whether two mutations ***a*** and ***b*** occur simultaneously more often than expected, we again model the probability of their simultaneous occurrence using a beta distribution where:


$$\;\alpha =\sum_{n=1}^{N}X_{a,n}X_{b,n}\;\;$$

$$\begin{array}{rl} \;\beta \;= & N-\sum_{n=1}^{N}X_{a,n}-\sum_{n=1}^{N}X_{b,n}+\alpha -\sum_{n=1}^{N}Y_{a,n}-\sum_{n=1}^{N}Y_{b,n}\\ & +\sum_{n=1}^{N}Y_{a,n}Y_{b,n}. \end{array}$$

Alpha is the number of branches where both mutations occur, and beta is the number of branches where neither mutation occurs and neither mutation has already occurred.

The null expectation for the frequency with which mutations occur on the same branch is based on the individual frequency of the mutations:


$$E(a,b)=\frac{\sum_{n=1}^{N}X_{a,n}}{N-\sum_{n=1}^{N}Y_{a,n}}\times \frac{\sum_{n=1}^{N}X_{b,n}}{N-\sum_{n=1}^{N}Y_{b,n}}.$$

We then determine the probability of drawing the null expectation from the estimated distribution of co-occurrence probability, which constitutes the *P* value.

### Implementation of Dependency Tests

Tests were implemented using Python 3.9.13, using the beta distribution from statsmodels v.0.11.1 (Seabold and Perktold 2010). Pseudocounts of 1 were added to *β_M_*, *α_L_*, and *β_L_* to ensure validity of the beta distribution (*α_M_* is always at least 1 otherwise the mutation would not be tested). The *multipletests* function from statsmodels was used to implement the Benjamini–Hochberg correction with alpha 0.01.

### Evaluating Branch Lengths Between Dependent Mutations

For sequentially occurring dependent mutations, we sought to determine the median branch length separating occurrences of mutation *a* from occurrences of its preceding mutation *b*. We use ETE3 v3.1.2 to parse the phylogeny output by SNPPar to compute distances between the ancestor node of the branch where mutation *b* occurred, and the descendant node of the branch where mutation *a* occurred. This analysis was performed on a per-lineage-tree basis. If mutation *b* is inferred to be present on a particular lineage tree, but not to have occurred within the evolution of the lineage (i.e., occurred on the branch separating the ancestor of that lineage from the *M. tuberculosis* common ancestor), we designate the root note as the node where mutation *b* occurred. For each pair of mutations *a* and *b*, we take the median of the distance between all occurrences of *a* on background *b*.

Upon examination of the distribution of the median distance between dependent mutation pairs, we see that there is a small set (*N* = 49) of clear outliers with median distance > 1,000, which were removed from further analysis (supplementary fig. S4, Supplementary Material online).

### Assigning Mutations to Functional Categories

We define a set of known antibiotic resistance-associated sites based on World Health Organization data for 11 antituberculosis antibiotics: rifampicin, isoniazid, ethambutol, pyrazinamide, amikacin, kanamycin, capreomycin, streptomycin, levofloxacin, moxifloxacin, and ethionamide (W. H. (hq) Global Tuberculosis Programme 2021) (supplementary Data S5, Supplementary Material online). We also define a set of suspected resistance-associated sites, which includes all known resistance-associated sites, as well as any site in the same gene as a known resistance-associated site, and the entire intergenic regions upstream and downstream of each gene containing a resistance-associated site, using gene location data from Mycobrowser (Kapopoulou et al. 2011), because noncoding regions can have substantial effects on resistance phenotypes (Farhat, Freschi et al. 2019).

We define a set of antigenic genes based on data from immune epitope database (IEDB) (Vita et al. 2019). Following previous work on *M. tuberculosis* antigens (Coscolla et al. 2015), a list of all antigens was downloaded on January 7, 2022 with the following query criteria: linear peptide, organism = “*Mycobacterium tuberculosis* complex” (ID 77643), positive assays only, T-cell binding, any MHC restriction class, human host, any disease type, and any reference type. Any gene present in this list is considered antigenic.

To define gene essentiality, we take the union of all essential genes listed in Supplementary Table 3 of Minato et al. (2019), which summarizes the results of three studies on gene essentiality (Minato et al. 2019). We use two previous studies to define positions that contain lineage-associated variants (Freschi et al. 2021; Coll et al. 2014).

### Defining Resistance-Potentiating Mutations

We define any sequential mutation pair where the second mutation is a known resistance-conferring mutation and the first mutation is not as resistance potentiating. After observing that certain initial mutations occur before many different resistance-conferring mutations, we focus our analysis on these “most potentiating” initial mutations by selecting only those where the initial mutation is followed by over 30 different resistance-conferring mutations.

### GO Category Enrichment

We test whether our lists of dependent positions are enriched in particular GO categories. We downloaded the GO molecular function (MF) annotation for all 3,992 proteins in the *M. tuberculosis* proteome from pantherdb.org (Release 17.0, retrieved December 13, 2022) (Gene Ontology Consortium 2021; Mi et al. 2019; Ashburner et al. 2000), 2,558 of which have a GO MF annotation. For each GO MF, we compute the binomial *P* value of observing at least *k* hits to that GO MF category given that we observed *n* hits and that the current GO MF category has a frequency *p* among the 3992 proteins, using binom.cdf from SciPY v1.10.1 (Virtanen et al. 2020). We allow for multiple hits to the same protein (i.e., sampling with replacement) by counting each hit to a different nucleotide position. We do not count the same nucleotide position more than once. Multiple testing corrections were performed using the *multipletests* function of statsmodels v.0.14.0 (Seabold and Perktold 2010), implementing a Benjamini–Hochberg correction with alpha 0.01.

For testing whether the dependent mutations occurring after antibiotic resistance are enriched in any particular GO category, we have 1,987 hits to a unique nucleotide position, *n* = 1,622 of which are found in genes with a GO MF annotation. For testing whether dependent mutations not associated with antibiotic resistance or antigenic genes are enriched in any particular GO category, we have 3,724 hits to a unique nucleotide position, *n* = 2,744 of which occur in a gene with a GO MF annotation.

### Testing for Epistatic Effects of Dependent Mutations Using Linear Mixed Models

We measure which dependent mutations have a direct effect on antibiotic resistance, by running a series of linear mixed models of antibiotic MIC, including linear (additive) and interaction (epistatic) terms of each pair of variants (supplementary table S1, Supplementary Material online).

Association tests were run using GEMMA v0.98.1 using LMM mode and a missing allele threshold of 20% (Zhou and Stephens 2012). MIC data were obtained by combining data from multiple studies, for a total of 1,469 isolates (Farhat, Freschi, et al. 2019; Ezewudo et al. 2018; Eldholm et al. 2015; Phelan, Coll, McNerney, et al. 2016; Lee et al. 2014). For antibiotics tested in media other than 7h10, MIC values were normalized by dividing by the ratio of the critical concentration in 7h10 to the critical concentration in the tested media. MIC values were converted from a range to a number by taking the midpoint of the range, or the endpoint if only one point was provided (e.g., “>10” becomes “10,” and “2–4” becomes “3”), and then were log transformed. Alleles were encoded as 0 for ancestral state, 1 for nonancestral, or missing for positions where the allele could not be confidently called. Each evolutionarily dependent pair of sites was tested in a single multivariate linear mixed model, which included both sites as an independent term as well as an interaction term to capture epistatic effects. We controlled for population structure using a genetic relationship matrix (GRM) computed using all alleles (not just homoplastic variants) with a minor allele frequency greater than 0.1% across all 31,428 isolates in our data set.

### Computing Heritability

Heritability calculations were run using GEMMA v0.98.1 (Zhou and Stephens 2012), using the MIC values processed as described above. For each set of sites, the sites of interest were used to define a GRM, and the PVE by the GRM was calculated. This is equivalent to the heritability.

Three sets of sites were tested: 1) all homoplastic sites (sites with at least five independent mutations), 2) homoplastic mutations in a known or suspected resistance-conferring site, and 3) homoplastic mutations in a known or suspected resistance-conferring site, or found to be a dependent mutation with a mutation in a known resistance-conferring site (including both single and pair terms).

### Determining Physical Distance Between *nusG* and *rpoB* Mutations

We downloaded the solved structure of the RNA polymerase—NusG complex (PDB ID: 6z9p) (Said et al. 2021) from the RCSB PDB (Berman et al. 2000). We used MUSCLE from the EBI webserver with default parameters (Madeira et al. 2019) to align the sequence of *M. tuberculosis* NusG and RpoB to the sequence of the crystal structure. Then, we used PyMOL v2.4.0 to measure the physical distance between the residue corresponding to *M. tuberculosis* position 734624 (NusG R124), to any residue in the RpoB protein (Schrödinger LLC, 2015).

## Supplementary Material

msad131_Supplementary_Data

## Data Availability

All code is available on GitHub at https://github.com/farhat-lab/DependentMutations. Input and output data necessary for running the code and reproducing analyses in the paper are available on DataVerse, https://doi.org/10.7910/DVN/KMAACV. All strains used in our analyses are publicly available, and the raw read data are available for download from the NCBI using accession codes found in the isolate annotation table.
