# Supplementary material for: Analysis of Genome-Wide Mutational Dependence in Naturally Evolving Mycobacterium tuberculosis Populations
Source: Mol Biol Evol. 2023 Jun 23;40(6):msad131. doi: 10.1093/molbev/msad131 (PMC10292908; doi:10.1093/molbev/msad131)
Supplement: msad131_Supplementary_Data [file msad131_supplementary_data.zip › supplement_for_GreenAG_Dependent_Mutations.docx]

**Supplementary Material for:**

**Analysis of genome-wide mutational dependence in naturally evolving *Mycobacterium tuberculosis* populations**

Anna G. Green^1^, Roger Vargas Jr^1^, Maximillian G. Marin ^1^, Luca Freschi^1^, Jiaqi Xie^2^, Maha R. Farhat^1, 3^

**Affiliations**

^1^ Department of Biomedical Informatics, Harvard Medical School, Boston, MA, USA

^2^ Department of Genetics, Johns Hopkins School of Medicine, Baltimore, MD, USA

^3^ Division of Pulmonary and Critical Care Medicine, Massachusetts General Hospital, Boston, MA, USA

**Potts model results dominated by lineage variants and non-SNP mutational processes in *M. tuberculosis***

While Potts models have proven utility for detecting epistatic pairs in *Streptococcus pneumoniae, Streptococcus pyogenes, and Neisseria gonorrhoeae* (*1*–*3*), we find that in the particular case of *M. tuberculosis*, the results are dominated by putative non-SNP mutational processes and lineage-specific variants. Here, we define a non-SNP mutational process as any mutational event that generates more than one single nucleotide polymorphism at a time, either by substituting multiple bases or through recombination, which can manifest as apparent multiple substitutions after read mapping.

We initially ran a Potts model on all non-synonymous mutations with an allele frequency greater than 0.001, using the SuperDCA software package (*2*). To reduce the dataset size, we used only 11,015 isolates from our larger set of 31,428. We called variants relative to the reference genome of H37Rv, where any sites that show an alternative base in more than 40% of reads were called as polymorphisms. To further reduce dataset size while selecting mutations most likely to be functional, we translated the genome and selected only sites with non-synonymous variants, for a total of 36,489 sites. SuperDCA was run using a minor allele frequency threshold of 0.001 (for a total of 10,278 sites) and no-reweighting, with all other parameters set to default values. Following the SuperDCA workflow, we re-ranked the couplings based on phylogenetic weighting using HierBAPS(*2*, *4*). Top-ranking couplings were chosen by fitting a linear model where the couplings were used to predict the log10 of coupling rank, and selecting points with a residual greater than 5 times the estimated standard deviation, for a total of 201,789 significant pairs out of 42,980,356 (*1*, *2*) (**Supplementary Figure 1**). Despite following the literature standard procedure for phylogenetic weighting and determining significant hits, we found that the majority of the top-scoring hits were lineage-associated, and a substantial fraction were found in the same gene, potentially indicating evolution due to a single mutational event **(Supplementary Figure 2, Supplementary Table 9)**.

To better control for population structure, we then ran a Potts model on just the homoplastic sites in our analysis. Potts models were run on our set of 4,743 homoplastic variants from 31,428 isolates using the plmc package (*6*) with a maximum of 200 iterations. Alleles were encoded with three states: ancestral, derived, or gap (used for both deletions relative to the reference and uncertain allele calls). No sites in the alignment had more than 10% gaps. We scanned a range of theta values (0.01, 0.02, 0.05, 0.08, and 0.1) to determine a value which sufficiently corrects for oversampling of certain lineages without overcorrecting **(Supplementary Table 10)**. We chose theta = 0.02, which produces a N_eff of 3623.7. As in Schubert and Maddamsetti *et al*, we used a two-component mixture model to select the strongly coupled pairs, finding 31,753 pairs (out of a total of 11,255,140) over the 99% probability threshold (*3*, *6*). Couplings were processed using the EVCouplings Python package (*7*). To further control for population structure, we removed any pairs with a member in a lineage-associated position (*8*, *9*), leaving 26,873 pairs.

We find that while this protocol did correct for lineage-associated mutations, the model still has results dominated by mutations due to a single mutational event (**Supplementary Figure 3**). The top pairs tended to be in close genetic proximity – 98% of the top 500 hits are within 100 base pairs on the genome. While this may be explained by true evolutionary dependencies due to shared function of proximal base pairs, it may also be explained by multi-base mutations or intrachromosomal recombination (gene conversion). The ancestral sequence reconstruction analysis supported the latter possibilities: 52% of the top 500 pairs are predicted to arise on the exact same phylogenetic branch more than 80% of the time, and a further 14% are found in positions with more than 5% gaps, indicating possible insertions and deletions (**Supplementary Table 11**). The enrichment for non-SNP evolutionary events is potentially due to the focus of Potts models on isolate genomes: mutations that affect multiple sites manifest as multiple SNPs that always co-occur and are never found independently, generating strong signal.

**Supplementary Figure 1: Using a semi-log linear model to determine significant couplings from SuperDCA**

**Supplementary Figure 2: The top-scoring hits from SuperDCA are lineage-associated or single mutational event changes.** The top 10,000 ranked hits from SuperDCA, colored by their category, are shown. Mutation pairs are defined as “in lineage” if at least one mutation is lineage-associated according to HierBAPS. Mutation pairs that are not “in lineage” or found in the same gene are considered “standard”. Only the top 10,000 hits out of 201,789 are shown for visualization purposes.

**Supplementary Figure 3: Identity of top 500 Potts model hits among homoplastic sites.** The identity of the top-ranking pairs of couplings output by the Potts model. Sites are labelled as “standard” if they are not inferred to be part of the same mutational event and neither of the sites have >5% gaps in the input sequence alignment.


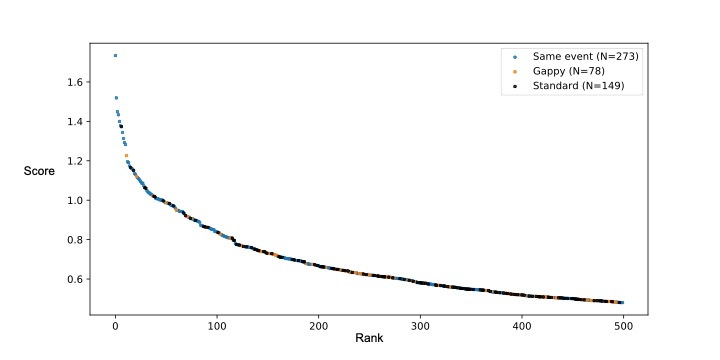


**Supplementary Figure 4: Distribution of branch lengths in dataset**

(A) Histogram of branch lengths for the six lineages of *M. tuberculosis* used in this study. Branch lengths are shown as number of substitutions (plus one, for log scaling). Most branches (72%) have ten or fewer mutations, and 99% have fewer than one hundred mutations. (B) Histogram of number of mutations (log scale) separating significant sequential dependent mutations in our dataset.

**
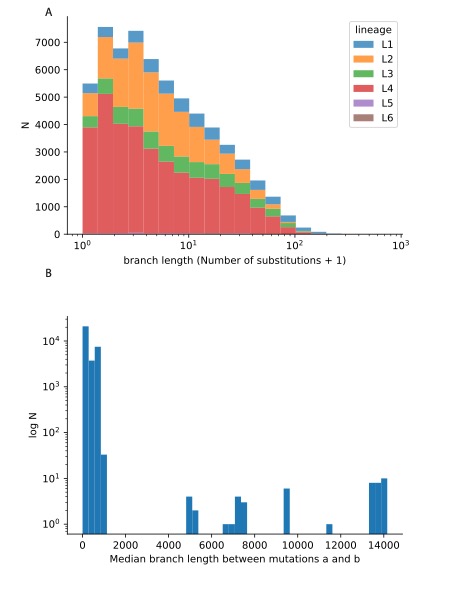
**

**Supplementary Figure 5: Properties of simultaneous, close-proximity mutations suggest non-SNP mutational processes.** (A) Number of simultaneous, dependent mutation pairs occurring at close genomic proximity, with bin size of 100 base pairs. The greatest enrichment in number of pairs is at distances of 0 to 100 base pairs, but enrichment continues up to 600 base pairs in distance. (B) Scatterplot and (C) Kernel density estimate of genomic distance of mutation pairs versus the fraction of times that mutation pair occurs simultaneously (versus independently or sequentially). A frequency of 1 means that the less common of the two mutations in the pair always occurs at the same time as the other mutation.**
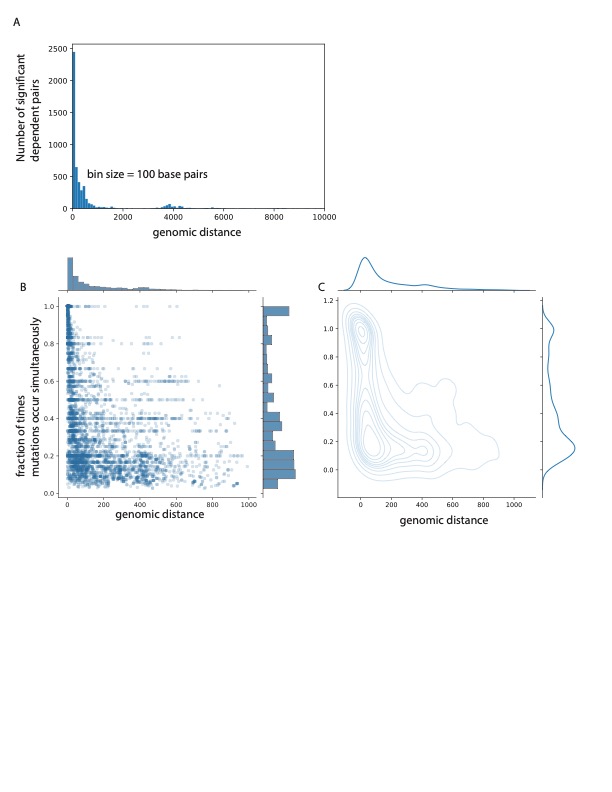
**

**Supplementary Figure 6: Sequential ordering of antibiotic resistance evolution capture by dependent mutations.** All sequential dependent mutations occurring between known antibiotic resistance mutations were enumerated, and the total number of occurrences of each were summed. Only sequential mutations were used because the ordering of simultaneous mutations cannot be resolved.

**Supplementary Table 1: Percent of known and dependent mutations with significant effect on MIC**

|  | Known resistance variants | | | Dependent mutations | | |
| --- | --- | --- | --- | --- | --- | --- |
| drug | Number tested | Number significant | Percent significant | Number tested | Number significant | Percent significant |
| AMIKACIN | 11 | 1 | 9% | 157 | 16 | 10% |
| CAPREOMYCIN | 9 | 4 | 44% | 136 | 38 | 28% |
| ETHAMBUTOL | 11 | 3 | 27% | 221 | 17 | 8% |
| ETHIONAMIDE | 24 | 3 | 13% | 95 | 7 | 7% |
| ISONIAZID | 7 | 3 | 43% | 270 | 8 | 3% |
| KANAMYCIN | 10 | 3 | 30% | 142 | 26 | 18% |
| MOXIFLOXACIN | 9 | 6 | 67% | 92 | 27 | 29% |
| PYRAZINAMIDE | 88 | 3 | 3% | 232 | 6 | 3% |
| RIFAMPICIN | 22 | 6 | 27% | 388 | 14 | 4% |
| STREPTOMYCIN | 32 | 5 | 16% | 317 | 36 | 11% |

**Supplementary Table 2: Consequential mutations with significant effect on MIC**

Mutations occurring after antibiotic resistance (consequential mutations) that have a significant influence on MIC, either linearly or epistatically. Variants and genomic positions indicated with a ‘+’ are epistatic interactions between the two variants that are found to be significant. Genes indicated as X-Y are found in the intergenic region between genes X and Y.

| Gene(s) | rs | drug | beta | p_wald |
| --- | --- | --- | --- | --- |
| hadA | 732110 | KANAMYCIN | 1.1613 | 0.0080 |
| rrs + hadA | 732110 + 1473246 | KANAMYCIN | 1.7481 | 0.0094 |
| Rv0029 + tlyA | 32411 + 1918634 | CAPREOMYCIN | 1.7937 | 0.0000 |
| purL | 895936 | CAPREOMYCIN | 2.1907 | 0.0003 |
| Rv0029 | 32411 | CAPREOMYCIN | 1.2861 | 0.0018 |
| p_inhA + inhA | 1674263 + 1673425 | ETHIONAMIDE | 0.8063 | 0.0018 |
| inhA + PPE19 | 1532777 + 1674481 | ETHIONAMIDE | 0.9874 | 0.0022 |
| p_inhA + inhA | 1674782 + 1673425 | ETHIONAMIDE | 2.1947 | 0.0032 |
| inhA | 1674782 | ETHIONAMIDE | 1.3317 | 0.0086 |
| katG + PE_PGRS28-Rv1453 | 1638364 + 2155168 | ISONIAZID | 1.0824 | 0.0000 |
| KatG + cspA-Rv3649 | 4088558 + 2155168 | ISONIAZID | -5.2103 | 0.0001 |
| cspA-Rv3649 | 4088558 | ISONIAZID | -5.2018 | 0.0001 |
| katG + Rv1873 | 2123182 + 2155168 | ISONIAZID | 1.3720 | 0.0002 |
| katG + vapC25 | 332951 + 761110 | RIFAMPICIN | 1.5771 | 0.0008 |
| embB + PE_PGRS28-Rv1453 | 1638364 + 4247429 | ETHAMBUTOL | 0.2971 | 0.0021 |
| p_embB | 4243217 | ETHAMBUTOL | 0.3911 | 0.0052 |

1. M. J. Skwark, N. J. Croucher, S. Puranen, C. Chewapreecha, M. Pesonen, Y. Y. Xu, P. Turner, S. R. Harris, S. B. Beres, J. M. Musser, J. Parkhill, S. D. Bentley, E. Aurell, J. Corander, Interacting networks of resistance, virulence and core machinery genes identified by genome-wide epistasis analysis. *PLoS Genet.* **13**, e1006508 (2017).

2. S. Puranen, M. Pesonen, J. Pensar, Y. Y. Xu, J. A. Lees, S. D. Bentley, N. J. Croucher, J. Corander, SuperDCA for genome-wide epistasis analysis. *Microb Genom*. **4** (2018), doi:10.1099/mgen.0.000184.

3. B. Schubert, R. Maddamsetti, J. Nyman, M. R. Farhat, D. S. Marks, Genome-wide discovery of epistatic loci affecting antibiotic resistance in Neisseria gonorrhoeae using evolutionary couplings. *Nature Microbiology*. **4**, 328–338 (2019).

4. L. Cheng, T. R. Connor, J. Sirén, D. M. Aanensen, J. Corander, Hierarchical and spatially explicit clustering of DNA sequences with BAPS software. *Mol. Biol. Evol.* **30**, 1224–1228 (2013).

5. C. Weinreb, A. J. Riesselman, J. B. Ingraham, T. Gross, C. Sander, D. S. Marks, 3D RNA and Functional Interactions from Evolutionary Couplings. *Cell*. **165**, 963–975 (2016).

6. A. Toth-Petroczy, P. Palmedo, J. Ingraham, T. A. Hopf, B. Berger, C. Sander, D. S. Marks, Structured States of Disordered Proteins from Genomic Sequences. *Cell*. **167**, 158-170.e12 (2016).

7. T. A. Hopf, A. G. Green, B. Schubert, S. Mersmann, C. P. I. Schärfe, J. B. Ingraham, A. Toth-Petroczy, K. Brock, A. J. Riesselman, P. Palmedo, C. Kang, R. Sheridan, E. J. Draizen, C. Dallago, C. Sander, D. S. Marks, The EVcouplings Python framework for coevolutionary sequence analysis. *Bioinformatics*. **35**, 1582–1584 (2019).

8. L. Freschi, R. Vargas Jr, A. Husain, S. M. M. Kamal, A. Skrahina, S. Tahseen, N. Ismail, A. Barbova, S. Niemann, D. M. Cirillo, A. S. Dean, M. Zignol, M. R. Farhat, Population structure, biogeography and transmissibility of Mycobacterium tuberculosis. *Nat. Commun.* **12**, 6099 (2021).

9. F. Coll, R. McNerney, J. A. Guerra-Assunção, J. R. Glynn, J. Perdigão, M. Viveiros, I. Portugal, A. Pain, N. Martin, T. G. Clark, A robust SNP barcode for typing Mycobacterium tuberculosis complex strains. *Nat. Commun.* **5**, 4812 (2014).

10. X. Zhou, M. Stephens, Genome-wide efficient mixed-model analysis for association studies. *Nat. Genet.* **44**, 821–824 (2012).

11. M. R. Farhat, L. Freschi, R. Calderon, T. Ioerger, M. Snyder, C. J. Meehan, B. de Jong, L. Rigouts, A. Sloutsky, D. Kaur, S. Sunyaev, D. van Soolingen, J. Shendure, J. Sacchettini, M. Murray, GWAS for quantitative resistance phenotypes in Mycobacterium tuberculosis reveals resistance genes and regulatory regions. *Nat. Commun.* **10**, 2128 (2019).

12. V. Eldholm, J. Monteserin, A. Rieux, B. Lopez, B. Sobkowiak, V. Ritacco, F. Balloux, Four decades of transmission of a multidrug-resistant Mycobacterium tuberculosis outbreak strain. *Nat. Commun.* **6**, 7119 (2015).

13. J. Phelan, F. Coll, R. McNerney, D. B. Ascher, D. E. V. Pires, N. Furnham, N. Coeck, G. A. Hill-Cawthorne, M. B. Nair, K. Mallard, A. Ramsay, S. Campino, M. L. Hibberd, A. Pain, L. Rigouts, T. G. Clark, Mycobacterium tuberculosis whole genome sequencing and protein structure modelling provides insights into anti-tuberculosis drug resistance. *BMC Med.* **14** (2016), doi:10.1186/s12916-016-0575-9.

14. J. Lee, D. T. Armstrong, W. Ssengooba, J.-A. Park, Y. Yu, F. Mumbowa, C. Namaganda, G. Mboowa, G. Nakayita, S. Armakovitch, G. Chien, S.-N. Cho, L. E. Via, C. E. Barry 3rd, J. J. Ellner, D. Alland, S. E. Dorman, M. L. Joloba, Sensititre MYCOTB MIC plate for testing Mycobacterium tuberculosis susceptibility to first- and second-line drugs. *Antimicrob. Agents Chemother.* **58**, 11–18 (2014).

15. M. Ezewudo, A. Borens, A. Chiner-Oms, P. Miotto, L. Chindelevitch, A. M. Starks, D. Hanna, R. Liwski, M. Zignol, C. Gilpin, S. Niemann, T. A. Kohl, R. M. Warren, D. Crook, S. Gagneux, S. Hoffner, C. Rodrigues, I. Comas, D. M. Engelthaler, D. Alland, L. Rigouts, C. Lange, K. Dheda, R. Hasan, R. McNerney, D. M. Cirillo, M. Schito, T. C. Rodwell, J. Posey, Integrating standardized whole genome sequence analysis with a global Mycobacterium tuberculosis antibiotic resistance knowledgebase. *Sci. Rep.* **8**, 15382 (2018).

16. K. Winther, J. J. Tree, D. Tollervey, K. Gerdes, VapCs of Mycobacterium tuberculosis cleave RNAs essential for translation. *Nucleic Acids Res.* **44**, 9860–9871 (2016).
